# Structural biology of microbial gas vesicles: historical milestones and current knowledge

**DOI:** 10.1042/BST20230396

**Published:** 2024-02-08

**Authors:** Stefan T. Huber, Arjen J. Jakobi

**Affiliations:** Department of Bionanoscience, Kavli Institute of Nanoscience, Delft University of Technology, 2629 HZ Delft, The Netherlands

**Keywords:** biomolecular assemblies, gas vesicles, GvpA, motility

## Abstract

Gas vesicles mediate buoyancy-based motility in aquatic bacteria and archaea and are the only protein-based structures known to enclose a gas-filled volume. Their unique physicochemical properties and ingenious architecture rank them among the most intriguing macromolecular assemblies characterised to date. This review covers the 60-year journey in quest for a high-resolution structural model of gas vesicles, first highlighting significant strides made in establishing the detailed ultrastructure of gas vesicles through transmission electron microscopy, X-ray fibre diffraction, atomic force microscopy, and NMR spectroscopy. We then survey the recent progress in cryogenic electron microscopy studies of gas vesicles, which eventually led to a comprehensive atomic model of the mature assembly. Synthesising insight from these structures, we examine possible mechanisms of gas vesicle biogenesis and growth, presenting a testable model to guide future experimental work. We conclude by discussing future directions in the structural biology of gas vesicles, particularly considering advancements in AI-driven structure prediction.

## Pioneering studies on gas vesicle structure

Gas vesicles are intracellular, gas-filled nanostructures that allow prokaryotes to float vertically to the surface of their aqueous habitat. Gas vesicles are unique among prokaryotic motility systems in that their structure alone imparts the function of motility. Aquatic cyanobacteria, for example, utilise this form of motility to optimise light harvesting conditions for photosynthesis by rising to the surface. Massive accumulation of such cyanobacterial aggregates can be seen in freshwater lakes, colloquially called algal blooms.

**Figure 1.**
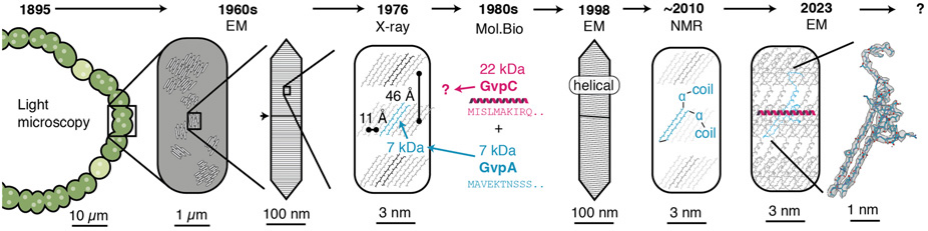
Historical milestones of investigating gas vesicle structure. This timeline charts the development from 19th century light microscopy to atomic structure determination. It highlights key findings and adopts an open-ended perspective, drawing attention to persisting unresolved structural questions discussed in the final section of this review.

The presence of gas vesicles reduces the density of cells and prevents them from sinking; ∼3–10% of the cell volume must be taken up by gas vesicles for a cell to float [1]. While the function of gas vesicles is straightforward — enabling buoyancy — the molecular architecture required to fulfil this role is complex and intriguing. Unravelling the structural complexity of these organelles poses a unique scientific challenge and has fuelled multiple waves of research employing a diverse set of structural and biophysical methods. All these contributions have furthered knowledge of structural and functional aspects of gas vesicles, yet each has also raised new questions warranting further study (Figure 1). In the following, we aim to trace the scientific trajectory that eventually led to a molecular model of gas vesicles at atomic detail, recapitulating the evolution of knowledge surrounding gas vesicle structure.

Bright, refractile bodies in cyanobacteria were first described in 1895 using light microscopy and identified as crucial components imparting buoyancy in these cells [2]. The gas-filled nature of these ‘Gasvacuolen’ (engl. gas vacuoles) was established [3] and their sensitivity to pressure was shown experimentally [4]. These early studies established the function of these intracellular gas spaces. Yet, their sub-micrometre dimensions hindered detailed structural analysis with the existing methodologies.

How can a cell produce and maintain a gas-filled structure? The increased availability of commercial electron microscopes in the 1960s [5] enabled the first detailed ultrastructural examination of gas vacuoles. These turned out to be gas-filled cylindrical objects, initially referred to as ‘Hohlspindeln’ (engl. hollow spindles) [6] with ‘gas vesicles’ becoming the widely accepted nomenclature today. Employing diverse electron microscopy techniques, seminal studies by Jost [6], Bowen and Jensen [7], Smith and Peat [8], and Walsby and Eichelberger [9], provided comprehensive insights into the overall morphology of gas vesicles. These studies established important ultrastructural features, including the cylinder structure with conical ends, the overall dimensions with diameters of 65–75 nm and lengths of 200–1000 nm (representative for cyanobacterial gas vesicles), the intracellular packing of gas vesicles into hexagonal clusters, the gas-filled central lumen, and more detailed features such as the periodic 4–5 nm spacing of surface ribs and a wall thickness of ∼2 nm. While the overall ultrastructure of gas vesicles is conserved across different phyla, the typical width and length distributions can differ substantially [1,10–12].

What molecular building blocks are gas vesicles composed of? Initially, the molecular composition of gas vesicles was unclear. Walsby and Buckland [13] developed methods to isolate gas vesicles from *Anabaena flos-aquae* cells with mild cell lysis and controlled centrifugation. Purified gas vesicles also allowed to measure pressure-collapse curves for assessing the vesicles’ stability and helped improving yield of intact gas vesicles by identifying protocols that limit hydrostatic pressure. Alongside a similar study on *Microcystis aeruginosa* [14], these efforts demonstrated that purified gas vesicles are exclusively made from protein, devoid of other biomolecular material such as lipids or carotenoids. The highly intriguing concept of protein assemblies forming extended gas-filled compartments inspired additional studies to unravel their exact composition and structure.

How can protein-based and gas-filled nanocompartments self-assemble inside the cell? Waaland and Branton [15] made key observations on the biogenesis of gas vesicles in 1969, noting a unique central rib in the gas vesicle wall and characterising the growth pattern of these vesicles over time. Gas vesicles seemed to initiate as small biconical structures, growing from the middle outwards by elongating the cylindrical centre. Walsby [16] suggested that this central rib results from the union of two identical halves of the gas vesicle, with unique molecular interactions occurring at this juncture. Examining the repetitive structural unit added during gas vesicle growth, Blaurock and Walsby [17] employed X-ray fibre diffraction to analyse gas vesicles from *A. flos-aquae*. Their findings indicated gas vesicles consist of a crystalline wall structure that is composed of 11 Å wide subunits, each 46 Å high and 20 Å thick, periodically arranged along the gas vesicle ribs. From the volume of this subunit the molecular mass was estimated to be ∼8 kDa. Diffractograms showed strong 4.7 Å peaks, consistent with the canonical distance of neighbouring β-strands in a β-sheet assembly. Along with the angular orientation found at a 35° tilt relative to the long axis of the vesicle and the overall subunit dimensions deduced from the fibre diffraction patterns, this led to the suggestion that gas vesicles are formed by tilted β-sheets consisting of staggered, horizontally aligned β-hairpins. Identifying the protein capable of forming this unique quasi-crystalline assembly became imperative with the establishment of the principal structural characteristics of the essential subunit.

## The molecular constituents GvpA and GvpC

During the 1980s, attempts to determine the complete sequence of the gas vesicle wall protein faced challenges due to its insolubility in SDS, complicating separation by gel electrophoresis. Despite these obstacles, partial sequences of the gas vesicle wall protein were identified through analysis of tryptic digests of purified gas vesicles from *A. flos-aquae* [18,19]. However, discrepancies emerged: a recurring band observed by SDS–PAGE indicated a molecular mass of 20.6 kDa [18], inconsistent with the 8 kDa estimated from X-ray fibre diffraction. Parallel efforts by De Marsac [20] supported by the partial sequences from Walsby, led to the cloning of a gas vesicle gene from *Calothrix* PCC7601, a cyanobacterium highly similar to *A. flos-aquae*, and revealed the complete genetic sequence of GvpA encoding a protein of 7.3 kDa. Subsequent protein sequencing from *A. flos-aquae* gas vesicles corroborated these findings, identifying a 70-residue protein (excluding the initial methionine) with an equivalent mass of 7.3 kDa [21], which also matched the earlier crystallographic data [17].

Initially hypothesised as a GvpA trimer, the ∼20 kDa band on SDS–PAGE gels from *A. flos-aquae* gas vesicles [21] was later attributed to a second structural gas vesicle protein, GvpC, with a molecular mass of 22 kDa [22,23]. Further investigation showed that detergent treatments could remove GvpC without compromising the overall morphology of the gas vesicle envelope, resulting in reduced stability to externally applied pressure and suggesting a vital role for GvpC in reinforcing the gas vesicle wall [24]. The experimental determination of the GvpA to GvpC ratio of 25:1 [25] in mature gas vesicles prompted proposals of various binding geometries in which GvpC either aligns along or spans across the gas vesicle ribs. While genetic and biochemical studies have elucidated the sequence, identity, and relative abundance of GvpA and GvpC in purified gas vesicles, they have also raised intriguing questions about their fold and three-dimensional (3D) structure. The geometric restraints on the asymmetric unit inferred from the X-ray fibre diffractograms indicated that the primary sequence pattern of GvpA is inconsistent with a subunit consisting entirely of β-strands [21]. Atomic force microscopy (AFM) provided images of the wall ultrastructure in collapsed gas vesicles at a level of detail not achievable with electron microscopy at the time. AFM estimates of rib periodicities (46.4 Å) and subunit repeats along the rib (11.2 Å), and the direct visualisation of β-strands spaced 4.7 Å apart were consistent with those of the earlier fibre diffraction patterns [26]. Intriguingly, AFM revealed gaps between the gas vesicle ribs, suggesting the need for a bridging element and leaving important questions about how successive ribs are held together.

Despite these advances on understanding gas vesicle ultrastructure, one central structural question about gas vesicles persisted. Since the 1960s, gas vesicles had been described as ribbed structures with rib periodicities ranging from 4.5 to 5 nm. Two competing models, stacked hoop aggregates or a continuous helix, were proposed [1,16]. Conclusive evidence for the helical model emerged from cryogenic electron microscopy (cryo-EM) experiments on frozen-hydrated gas vesicles from *Halobacterium salinarum* [12]. This approach helps preserve gas vesicles in a more native state, avoiding the structural flattening in dried, metal-stained samples. The moiré patterns, resulting from interference between the gas vesicle's front and back ribs, definitively established that the gas vesicle wall is formed by a continuous helical rib, not stacked hoops.

## New techniques: NMR and cryo-EM

Solid-state NMR spectroscopy, different in its underlying physical principles from X-ray diffraction and electron microscopy, contributed decisively to revealing GvpA's definite secondary structure. Resonance assignment of 81% of the GvpA sequence [27] uncovered a coil-α-β-β-α-coil pattern. This pattern, with 27 out of 71 residues forming the central β-hairpin and flanked by α-helices and coils, corroborated prior hypotheses based on sequence analysis [21] and AFM data [26]. Furthermore, the NMR studies indicated the existence of inequivalent GvpA monomers in the gas [28,29], suggesting these monomers were arranged in antiparallel orientations with different folds within the ribs. This hypothesis also influenced computational models of GvpA [30,31]. However, subsequent high-resolution structures eventually invalidated this antiparallel model. While conjointly, research over the past four decades had revealed important clues on the periodicities, the unit cell size, the molecular constituents GvpA and GvpC, and the secondary structure of GvpA, the challenge of deducing the precise 3D conformation of GvpA in the gas vesicle wall remained and with it a molecular-level explanation of the unique properties of gas vesicles. Gas vesicles can neither be crystallised into 3D crystals for X-ray crystallography, nor could their 3D structure be determined by NMR spectroscopy. The past decade saw dramatic technical improvements in cryo-EM with the introduction of better electron detectors, microscopes, and algorithms [32]. These advancements have enabled the direct determination of the 3D structures of macromolecular assemblies from two-dimensional (2D) particle images and offered a route to finally determine the 3D structure of the gas vesicle wall by direct imaging.

Pioneering efforts in using cryo-EM to elucidate the gas vesicle structure were undertaken by Bollschweiler [10] using a variant of cryo-EM called cryogenic electron tomography (cryo-ET). This study focused on an I34M GvpA mutant from the halophilic archaeon *H. salinarum*, known for producing thinner and more homogeneous gas vesicles compared with the wild-type strain [31]. A key observation was the extreme sensitivity of gas vesicles to electron exposure, with structural degradation occurring even at very low fluence. To mitigate radiation damage, tilt series were acquired using only a fraction of the typical electron exposure, allowing the use of subtomogram averaging to obtain a reconstruction of the wall structure at an approximate resolution of ∼9 Å. The analysis revealed hook-like features in the wall leading to a corrugated outline with a reversal in orientation at the central rib of the gas vesicle located at the midpoint along its long axis, all while maintaining constant helical handedness in both cylinder sections. However, the obtained resolution was not sufficiently high to discern the secondary structural elements of GvpA, specifically the coil-α-β-β-α-coil pattern predicted from NMR, thus leaving the precise fold of GvpA unresolved.

A more recent study focused on cryo-ET structure determination of gas vesicles from *A. flos-aquae* [33], which have smaller diameter and greater stability compared with those in *H. salinarum*. Together with advances in cryo-ET technology over the preceding eight years, this facilitated reconstructing the gas vesicle wall at a slightly better resolution of 8 Å. At this resolution, the sub-averaged tomographic reconstruction revealed a continuous band of density along the rib, interpreted as the polymerising β-strands of GvpA monomers. These β-strands and adjacent densities confirmed the corrugated pattern in the wall's cross-section. Moreover, the resolved density provided the first interpretable insight into the ultrastructure of the GvpC binding geometry to the exterior wall of the gas vesicle, establishing its alignment along the ribs.

Both cryo-ET studies highlighted a common challenge in resolving molecular details of gas vesicles using electron imaging methods: the resolution along the gas vesicle ribs was much poorer compared with other spatial directions. This difficulty stems from the lack of large-scale features along the gas vesicle rib, which are crucial for particle alignment and averaging. The rib structure is primarily characterised by a 4.7 Å spacing between β-strands of GvpA and, to a lesser extent, by a 12 Å spacing between the GvpA monomers. At resolutions below 12 Å, no distinctive features along the ribs are discernible. Alignment of these 4.7 and 12 Å structural features is particularly challenging under conditions of low signal-to-noise ratio (SNR) caused by the limitations on electron exposure required to mitigate radiation damage [10] and gas vesicle shrinking [33]. This is exacerbated by other challenges of cryo-ET, such as imperfect tilt series alignment, increased sample thickness at high tilt angles and thick ice layers due to the properties of the sample itself. These conditions impede resolving structural features beyond the overall ultrastructure of the gas vesicle wall and lead to anisotropic reconstructions with less detail along the gas vesicle ribs.

## Atomic structure of the gas vesicle wall

**Figure 2.**
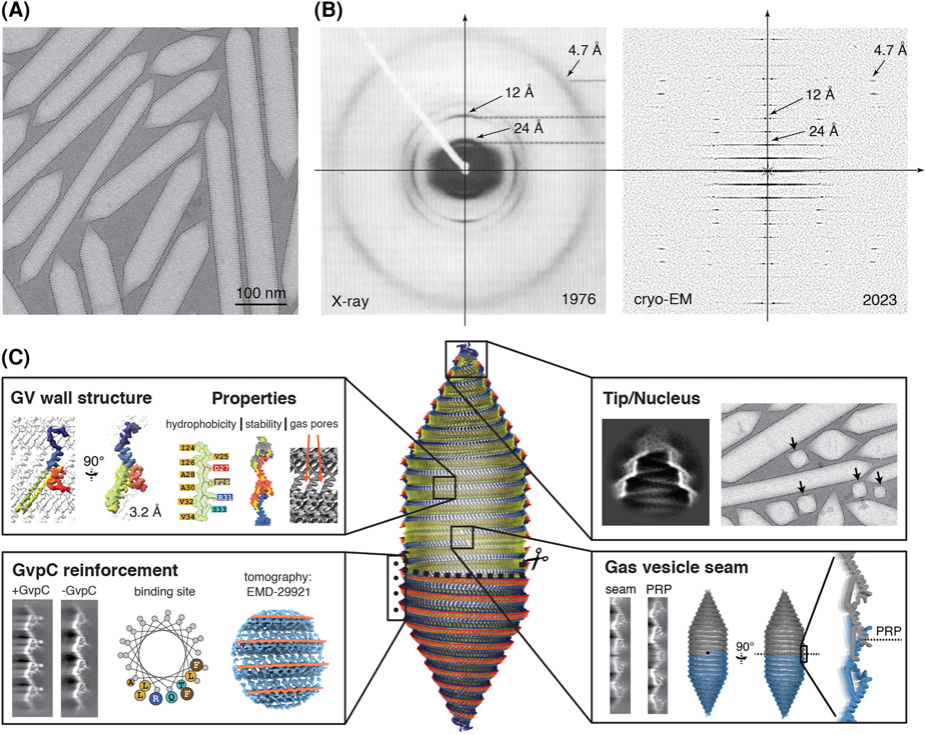
Atomic structure of gas vesicles. (**A**) Cryo-EM image of a *B. megaterium* gas vesicle monolayer embedded in thin vitreous ice. (**B**) Fibre diffraction patterns of aligned gas vesicles (from Blaurock and Walsby [17], reproduced with the consent of the publisher), compared with the sum of Fourier amplitude spectra from aligned cryo-EM images of gas vesicles [35] illustrating technical advances over the last 50 years and the associated improvement in data quality that allowed determination of the atomic structure of gas vesicles. (**C**) Detailed structure of gas vesicles, showing a pseudo-atomic model of an entire vesicle, the atomic structure of GvpA forming the gas vesicle wall and the resulting physicochemical properties [35], the location and overall binding geometry of GvpC (from [35]; tomographic density from [33]), and the structural features of the gas vesicle seam and tip [35].

To circumvent the experimental challenges inherent to tomographic imaging of gas vesicles we opted for 2D imaging over tilt-series acquisition, which allows for lower accumulated exposure to reduce electron beam- induced radiation damage. We prepared samples of *Bacillus megaterium* gas vesicles [34] in ultra-thin monolayers to maximise SNR. The 2D data obtained (Figure 2A), coupled with helical reconstruction techniques [36], enabled the first atomic structure determination of the gas vesicle envelope, including detailed molecular features along the GvpA ribs. The high-resolution signal present in the 2D cryo-EM data is evident when comparing 1976 X-ray fibre diffraction patterns [17] and amplitude spectra from Fourier transforms of 2023 electron micrographs [35] (Figure 2B), highlighting the technical advancements over the last few decades that ultimately made atomic structure determination possible. Using the approach outlined above, we determined the structure of the GvpA monomer in the context of the gas vesicle wall at 3.2 Å resolution [35]. This facilitated precise de novo atomic model building, finally unveiling the fold of the coil-α-β-β-α-coil secondary structure of GvpA, the amino acid side chain positions and orientations and the packing of the GvpA monomer within the gas vesicle rib. Our findings provide a comprehensive view of the gas vesicle's molecular architecture (Figure 2C), explaining its physical properties such as the extremely hydrophobic interior, corrugations leading to structural stability, and the selective permeability through its 3.8 Å gas pores [35]. The continuous, hydrophobic luminal surface forms an energetic barrier to condensation of water molecules, which along with structural pores enables selective transitioning of gas molecules across the wall by passive diffusion and thereby establishes the physical requirements for a stable gas-filled volume. The structure also confirmed the positioning of the auxiliary protein GvpC along the gas vesicle rib, consistent with cryo-ET observations [33]. Supporting bioinformatics analysis allowed determining the binding mode of GvpC to the α2 helix of GvpA through a series of conserved, periodically repeating arginine, leucine, and phenylalanine residues (Figure 2C).

## Structural unknowns: molecular mechanism of nucleation and growth

**Figure 3.**
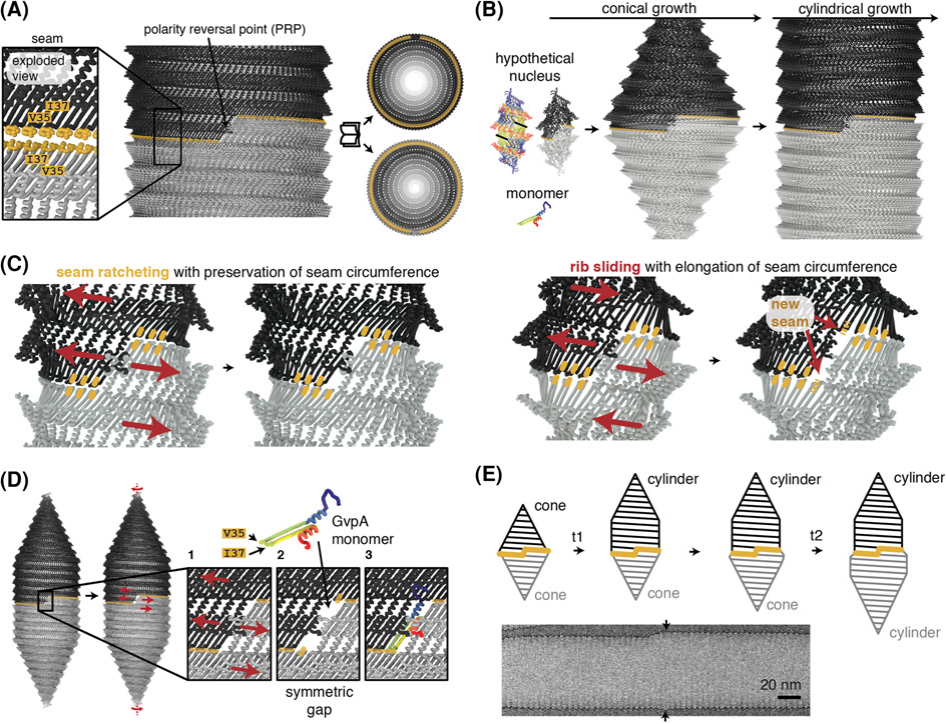
Model of gas vesicle biogenesis. (**A**) Molecular details of the gas vesicle seam formed by hydrophobic residues V35 and I37 of GvpA hairpins located on both gas vesicle halves pointing towards the seam. (**B**) Growth stages of gas vesicles. (**C**) Different growth mechanisms of cylindrical gas vesicle segments (where the seam needs to preserve its circumference) and for bicones (where the seam needs to increase in circumference). (**D**) Symmetric gap creation at the PRP allows monomer insertion for extension of the cylindrical section of the gas vesicle. The example shows insertion at the top half; insertion at the bottom half is geometrically equivalent. (**E**) Extended growth model highlighting independent cone-to-cylinder transitions for either half (t1 and t2) which leads to unequal diameters of cylindrical segments in both halves. Cryo-EM micrograph of *B. megaterium* gas vesicle with unequal cylinder diameters [35]. Arrows indicate the location of the seam.

Combining information of our atomic structure of GvpA and 2D projection images of the gas vesicle seam and tips, we constructed a complete model of the entire gas vesicle [35]. This model comprises two identical halves in reverse orientation joining at a ‘seam’ and confirms theoretical considerations initially postulated more than four decades earlier [16] (Figure 3A). The seam, formed by hydrophobic interactions between residues V35 and I37 of the contacting GvpA β-turns from both gas vesicle halves (Figure 3A) features a unique and critical element termed the ‘polarity reversal point’ (PRP). At the PRP, the helical ribs of both gas vesicle halves contact side-by-side, resulting in a reversal of the orientation of GvpA.

In the initial stages, gas vesicles grow as small biconical structures, potentially transitioning to cylindrical growth upon reaching a critical diameter. A hypothetical, elementary biconical nucleus might involve a single GvpA rib in each cone segment (Figure 3B). While cryo-EM has captured images of small bicones [35,37], the existence, formation process, and structural details of such an elementary nucleus remain speculative. Furthermore, it is yet unclear whether the bicone represents the initial nucleating structure of the gas vesicle or results from the combination of two smaller nuclei which form independently before merging into a bicone by assembling in opposing orientations. Isolating, enriching, and purifying these nascent stages of gas vesicle assembly would be pivotal for a deeper understanding of structure and composition during gas vesicle nucleation.

At a critical diameter, gas vesicle bicones continue to grow with constant diameter thus marking the transition to cylindrical growth. This critical diameter is not tightly controlled, and gas vesicles exhibit a range of cylinder diameters [11,35,38]. The mechanism underlying the cone-to-cylinder transition remains poorly understood. Based on structural considerations, it was proposed that the energetic balance between preferred curvature of GvpA and the binding energy gained by rib-to-rib contacts in an expanding cone could favour a cone-to-cyclinder transition at a certain critical diameter [35].

The GvpA N-terminus forms well-defined contacts with the β-turn of the adjacent rib in the crystalline, cylindrical wall segments, as compared with more disorganised contacts in the cone. Alterations in amino acids in the β-turn or N-terminus could thus influence gas vesicle morphology by modifying this energetic balance, as observed *H. salinarum* with mutations around the β-turn (e.g. I34M, corresponding to I37 in *B. megaterium* GvpA) [39] and the S13G mutation at the GvpA N-terminus of *H. salinarum* GvpA expressed from either the p-vac (plasmid-derived) or the c-vac (chromosomal) gene cluster, which results in spindle-shaped or cylindrical gas vesicles, respectively [12]. We have formulated a hypothesis for the cylindrical growth of gas vesicles, based on the assumption that there is a 1:1 binding of GvpA monomers from both sides of the seam [35]. This models centres on the creation of a symmetric gap at the PRP to integrate additional GvpA monomers. The rotational movement of one half of the gas vesicle relative to the other facilitates this gap creation, involving a ratcheting action with disruption and reformation of hydrophobic contacts between V35 and I37 at the seam (Figure 3C). A new GvpA monomer can then be incorporated into the gap, reforming the fully closed seam. Crucially, the total length of the seam remains unchanged in this model.

This seam ratcheting mechanism can explain how two contacting helical cylinders can grow but is incompatible with the growth of a bicone. In a growing bicone, the contacting bases increase in size, necessitating seam enlargement. We propose a different mechanism of growth for the bicone stage involving sliding of the ribs against each other within each cone while the seam remains connected through its hydrophobic contacts. [Supplementary Movie 1]. By this mechanism, the seam enlarges by one monomer, creating a similar opening at the PRP for monomer insertion (Figure 3C) as in the model for cylindrical growth. Such sliding between the gas vesicle ribs may be possible in the cone due to the lack of a crystalline relationship between the ribs as it occurs in the cylindrical segment.

The challenge with this model of bicone growth is that the seam grows by an entire GvpA monomer at each insertion step. In theory, if 100 monomers were added in an original cone of only 20 monomers, the resulting cone would have more than 100 monomers located at the seam (of the 120 monomers in total), which is geometrically impossible. The real growth mechanism of the bicone is likely more complex and may involve a mix of rib sliding and seam ratcheting. For both sliding and ratcheting mechanisms, a gap at the PRP allows for vesicle growth by insertion of new monomers. The PRP formed by contact of two gas vesicle halves in reverse orientation with 180-degree rotational symmetry relative to the long axis of the gas vesicle, makes it geometrically equivalent for a GvpA monomer to be inserted into either top or bottom gas vesicle halves (Figure 3D) and suggests that both halves can grow at random rates. Indeed, the seam is only approximately observed in the gas vesicle centre [33,35].

Unlike our idealised model, it has been observed that both gas vesicle halves can have unequal diameters [10,33,35]. This may be explained by the cone-to-cylinder transition not being tightly coupled and occurring independently in both halves (Figure 3E). The exact mechanism of gas vesicle growth therefore remains a highly intriguing problem in structural biology. Resolving the seam structure could yield deeper insight into the growth mechanism. Further understanding might also come from other biophysical techniques that allow monitoring gas vesicle growth kinetics in reconstituted systems.

## Structure and role of other gas vesicle gene products

**Figure 4.**
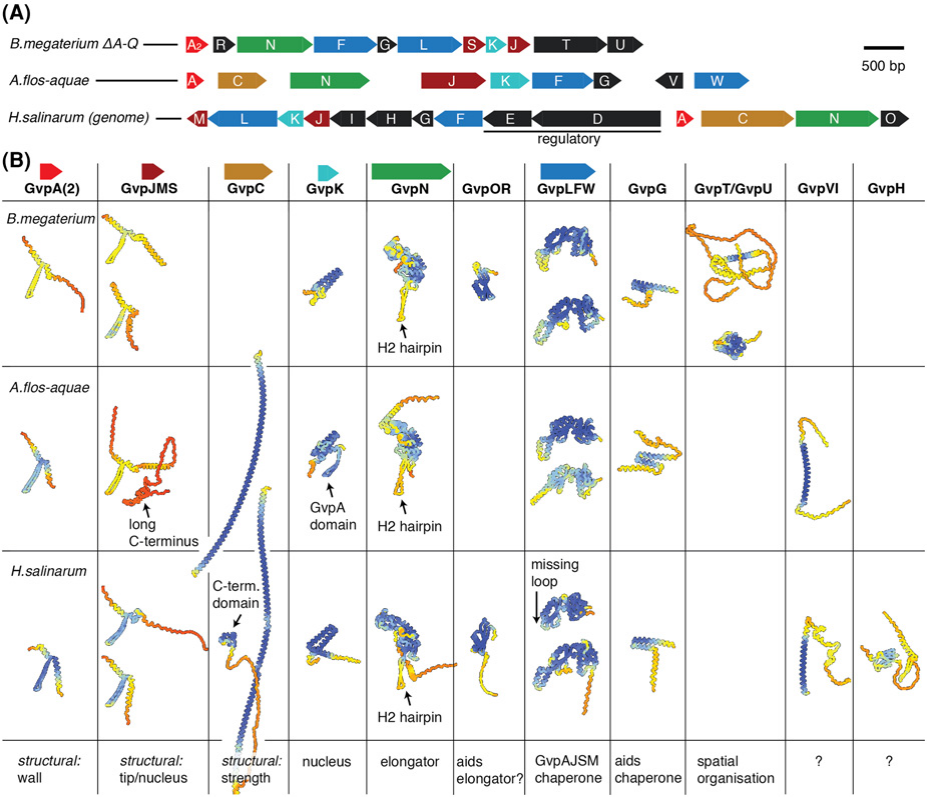
Protein structure predictions for gas vesicle gene clusters. (**A**) Gas vesicle gene clusters from three different organisms. (**B**) AlphaFold2 structure predictions for gene clusters from *B. megaterium*, *A. flos-aquae* and *H. salinarum*. All proteins are depicted to scale. GvpD and GvpE from *H. salinarum* were omitted due to their role in gene regulation [40]. The colour scheme highlights prediction confidence (blue: high, yellow: low). Arrows highlight noteworthy features. Annotation sources: GvpA [10,33,35], GvpG [41], GvpLFW [42,43], GvpOR [41], GvpK [41–43], GvpN [37,44], GvpJMS [37], GvpC [24], GvpTU [45].

Gas vesicles predominantly consist of the structural proteins GvpA and GvpC, yet the synthesis of these proteins requires a cluster of ten or more additional genes (Figure 4A). The functional roles of the genes within the clusters remain unclear. To shed light on this, we retrieved AlphaFold2 [46] predictions for gene products of the gas vesicle gene clusters from *B. megaterium*, *A. flos-aquae*, and *H. salinarum* and annotated them with evidence from recent biochemical studies for potential functional interpretation (Figure 4B). This presentation aims to serve as a hypothesis generator to fuel further research in order enhance our mechanistic understanding of gas vesicle biogenesis. We emphasise aspects that warrant further investigation.

Across all gene clusters, homologs of the major structural protein GvpA, such as GvpJ, GvpM, or GvpS are present, but their gene products are absent from the cylindrical part of the gas vesicle wall [35]. Recently they were identified in purified bicones [37], indicating their contribution to conical segment formation. Considering our earlier discussion on distinct growth mechanisms for cylindrical and bicone segments, these GvpA homologs likely play a crucial role in the unique growth processes hypothesised for bicones. Notably, the *A. flos-aquae* gene cluster is distinct, containing only a single GvpA homolog, GvpJ, which is further distinguished by an atypical C-terminus comprising 6 repeats of 21 amino acids, the functional significance of which remains unknown.

GvpC, a long α-helical protein characterised by repetitive sequences, stabilises gas vesicle ribs [24]. Cyanobacterial GvpC contains uniform 33 amino acid repeats that span four GvpA monomers along a rib, while haloarchaeal GvpC exhibits 32–38 amino acid repeats and includes a compact C-terminal domain connected to the α-helical repeats by an extended linker (Figure 4B). The specificity of GvpC for GvpA is associated with a characteristic motif comprising arginine, phenylalanine, and leucine clustering on one side of the α-helical repeat sequence [35]; however, the precise atomic structure of this binding interface remains undetermined. Furthermore, the pattern of GvpC's attachment to gas vesicles — whether random or regularly spaced — remains unclear, with indications of GvpC self-interaction from split-GFP assays [47] hinting at a more regular mode of binding.

GvpK, consistently found across gas vesicle gene clusters, is implicated in the initiation of gas vesicle formation [41–43], a role supported by its pronounced hydrophobicity suggested by AF2 predictions. Particularly noteworthy is the *A. flos-aquae* gene cluster where GvpK includes a GvpA-like domain, suggesting it may be a fusion combining aspects of GvpJMS with GvpK. This attribute renders it as an interesting target for studies of gas vesicle nucleation. Understanding the structure of an elementary nucleus could greatly advance our knowledge of initiation of gas vesicle assembly and the composition of the nucleus.

GvpN, an AAA + ATPase found in all gas vesicle gene clusters is essential for gas vesicle formation. While bicones can initially form without GvpN, they fail to transition from cone to cylinder or to elongate as cylinders [37,44]. Considering our earlier discussion on distinct growth mechanisms for cylindrical and bicone structures, GvpN may provide the energy required for seam ratcheting in the cylindrical growth phase. Intriguingly, GvpN features an atypically long 30 amino acid β-hairpin within its H2 insert [48] (Figure 4B), reminiscent of the extended GvpA β-hairpin, suggesting a potential functional interplay. Unravelling the role of GvpN and the underlying mechanism of its action remains a compelling challenge for structural studies on gas vesicle biogenesis.

Not surprising given its highly hydrophobic nature, GvpA has been shown to be associated with GvpF or GvpL, possibly acting as chaperones [41–43]. The interaction site between GvpF and GvpA, involving GvpA's α-1 helix [42] is suggestive of a co-translational chaperoning role during the protein synthesis process. Resolving the structure of GvpA in its chaperoned state is crucial for a comprehensive understanding of this interaction, as well as to discern possibly different structural states of GvpA when bound to a chaperone compared with that in its assembled form in the gas vesicle wall. The molecular mechanism underlying the solubilisation of GvpA in the cell and safely directing its integration into the gas vesicle assembly are compelling subjects for investigation. Likewise, the existence of a specific molecular mechanisms of gas vesicle degradation and if/how this process is regulated remains to be explored.

A recent study suggested functional roles for the proteins GvpU and GvpT, implicating them in inducing the aggregation of gas vesicles in cells by interacting with the elongated C-terminus of GvpA in *B. megaterium* [45]. Tight packing, or clustering, of gas vesicles has been previously observed in cyanobacteria by electron microscopy [6]. Notably, both the GvpU and GvpT genes are absent from cyanobacteria. They are exclusive to the phylum Bacillota (previously known as Firmicutes) and lack homologous counterparts in other phyla, suggesting that gas vesicle clustering may have evolved independently in phylogenetically distant organisms.

While recent high-resolution structures of gas vesicles mark the beginning of the quest to understand the assembly mechanism of these unique protein-based compartments, numerous unanswered questions persist. Structural studies targeting gas vesicle nuclei, tips, and seams, as well as the complex molecular machinery facilitating gas vesicle biogenesis will be pivotal for advancing our understanding of this unique form of prokaryotic motility.

## Perspectives

- Gas vesicles are protein assemblies that facilitate microbial motility through buoyancy control. Their simple, yet ingenious architecture ranks them among the most intriguing macromolecular assemblies known.
- Detailed structural knowledge has become available that explains the unique evolutionary adaptations that enable buoyancy-controlled motility in microbes.
- The detailed mechanisms underlying nucleation and elongation of gas vesicles remain elusive, as do the functions of numerous genes encoding the molecular machinery integral to gas vesicle biogenesis.

## Abbreviations

AFM: atomic force microscopy
cryo-EM: cryogenic electron microscopy
cryo-ET: cryogenic electron tomography
PRP: polarity reversal point
SNR: signal-to-noise ratio
